# A qualitative evaluation of a novel intervention using insight into tobacco industry tactics to prevent the uptake of smoking in school-aged children

**DOI:** 10.1186/s12889-016-3205-8

**Published:** 2016-07-11

**Authors:** John Taylor, Amy Taylor, Sarah Lewis, Ann McNeill, John Britton, Laura L. Jones, Linda Bauld, Steve Parrott, Qi Wu, Lisa Szatkowski, Manpreet Bains

**Affiliations:** UK Centre for Tobacco and Alcohol Studies, University of Nottingham, Division of Epidemiology and Public Health, Clinical Sciences Building, Nottingham City Hospital, Nottingham, NG5 1PB UK; UK Centre for Tobacco and Alcohol Studies, King’s College London, National Addictions Centre, Institute of Psychiatry, Psychology and Neuroscience, Addiction Sciences Building, 4 Windsor Walk, Denmark Hill, London SE5 8BB UK; UK Centre for Tobacco and Alcohol Studies, Public Health, Epidemiology and Biostatistics, School of Health and Population Sciences, College of Medical and Dental Sciences, University of Birmingham, Edgbaston, Birmingham B15 2TT UK; UK Centre for Tobacco and Alcohol Studies, Institute for Social Marketing, 3Y1, University of Stirling, Stirling, FK9 4LA UK; UK Centre for Tobacco and Alcohol Studies, Department of Health Sciences, University of York, Seebohm Rowntree Building, Heslington, York YO10 5DD UK

**Keywords:** Smoking prevention, Health education, Adolescents, Tobacco industry

## Abstract

**Background:**

Evidence from the US Truth campaign suggests that interventions focusing on tobacco industry tactics can be effective in preventing smoking uptake by children. Operation Smoke Storm is the first school-based intervention based on this premise and comprises three classroom sessions in which students act as secret agents uncovering tobacco industry tactics through videos, quizzes, discussions, and presentations. We report a qualitative evaluation of its acceptability.

**Methods:**

We conducted eight focus groups with 79 students aged 11-12 who participated in Operation Smoke Storm at two UK schools in Autumn 2013, and 20 interviews with teachers who delivered the intervention. These were digitally audio-recorded, transcribed verbatim and analysed using the framework method.

**Results:**

Students enjoyed the secret agent scenario and reported acquiring new knowledge about smoking and the tobacco industry, which seemed to strengthen their aversion to smoking. Teachers felt confident delivering the ‘off the shelf’ resource, although they would have welcomed more background information about the topic and guidance on steering discussions. Teachers highlighted a need for the resource to be flexible and not dependent on lesson length, teacher confidence, or expertise. Students and teachers endorsed the idea of developing a booster component for older students and supported the development of printed information complementing the resource to encourage parents to support their child not to smoke.

**Conclusions:**

These findings demonstrate that Operation Smoke Storm can be delivered by teachers to raise awareness about smoking-related issues. The ideas and issues raised are now being used to improve and extend the resource for further evaluation.

## Background

Globally, tobacco use kills nearly six million people each year, up to half of those who smoke [1]. In the UK, nearly 40 % of adult smokers start to smoke regularly before the age of 16 [2], and over 200,000 children start smoking each year [3]. Smokers who start at an early age smoke more cigarettes per day in adulthood [4], smoke for longer [5], are less likely to quit [6] and are more likely to die from smoking-related diseases [5]. Intervening with young people to prevent them from starting to smoke is therefore a public health priority.

School-based smoking prevention education is potentially a good way to reach large numbers of young people with an anti-smoking message. However, whilst existing evidence shows that school-based interventions to reduce the uptake of smoking may have short-term positive effects, there is little robust evidence that these interventions prevent young people from taking up smoking in the longer term [7–9].

There is little consensus as to whether any one approach to youth smoking prevention is superior. Existing education resources have concentrated on giving young people information on the harms of smoking, increasing their self-esteem and confidence, and teaching them skills to say ‘no’ if offered a cigarette [9]. In the UK, training young people as peer educators able to initiate conversations about smoking with their peers has been shown to reduce smoking uptake up to two years later [10]. Evidence from the US suggests that a focus on the ethics and exploitative tactics of the tobacco industry may be more effective in encouraging young people not to smoke [11, 12]. Though not specifically a school-based intervention, the US *Truth* campaign [13] exposes the tobacco industry’s deceptive marketing strategies, the addictive nature and health effects of cigarettes, and the negative effects of the industry on the environment and society.

The emphasis of the *Truth* campaign has been adopted by *Kick It*, the UK National Health Service Stop Smoking Service for Hammersmith and Fulham, Kensington and Chelsea, Westminster, Kingston upon Thames and Richmond upon Thames, in designing *Operation Smoke Storm* [14], a novel educational package for use in schools (see Table 1). Here we report a qualitative evaluation of the implementation of *Operation Smoke Storm* in two UK schools in preparation for a full-scale cluster-randomised controlled trial. To the best of our knowledge, this is the first time the approach of the *Truth* campaign has been tested in a school setting, either in the UK or internationally. The purpose of this initial small-scale implementation was to investigate the acceptability of *Operation Smoke Storm* with students and teachers and identify any improvements needed prior to wider evaluation. We have followed the COREQ guidelines for reporting qualitative studies [15].

**Table 1:** Operation Smoke Storm

*Operation Smoke Storm* is a web-based novel educational package designed to be delivered by teachers as part of a school’s Personal, Social, Health and Economic Education (PSHE) curriculum. Teachers are provided with detailed lesson plans for 3 x 50 minute classroom sessions (although the material can also be delivered as one longer session). Students act as secret agents to uncover the tactics of the tobacco industry and share what they find with others. The sessions also cover the health effects of tobacco, passive smoking, nicotine addiction and the economic cost of smoking.
Sessions one and two include video clips followed by individual and group-based quizzes, and discussion activities where students learn about the harmful and addictive nature of smoking and methods used by tobacco companies to encourage young people to smoke. Students are provided with a workbook to record their answers. In session three, they then use this information to ‘spread the word’ in a group presentation to their class, in a medium of their choice such as through drama or song.

## Method

### Delivery of operation smoke storm

Of six secondary schools approached in the UK East Midlands region, two agreed to participate in the delivery and evaluation of *Operation Smoke Storm.* Whilst the schools that declined our invitation expressed interest in the *Operation Smoke Storm* resource itself, they cited time pressures as their reason for not being able to participate. The two participating schools had contrasting socio-demographic profiles. School 1 was located in a market town, serving a relatively affluent catchment area in the town and surrounding villages. School 2 was in a small town on the edge of a major urban area. In School 1, 6.1 % of students were eligible for free school meals (a frequently-used measure of deprivation [16]) and in School 2, 10.2 %. Nationally, approximately 16 % of pupils are eligible to receive free school meals, with the figure reaching 75 % in some schools [17], and thus the two schools may not be representative of those in particularly deprived areas.

The research team provided a brief training session to teachers which outlined how to access and navigate the *Operation Smoke Storm* resource, and described the planned research-related activities. In total, 585 Year 7 (aged 11-12) students received *Operation Smoke Storm* during their usual PSHE lessons (School 1: 347 students in 14 classes; School 2: 238 students in 8 classes). School 1 had shorter lessons (40 minutes per week) and so some teachers took more than three sessions to cover the material. In School 2 PSHE was taught for 1 hour per fortnight; here delivery took place over three lessons, but there were wider intervals between the sessions.

### Study design and participant recruitment

Parents of Year 7 students were sent a letter informing them about *Operation Smoke Storm* and the accompanying academic evaluation, approximately three weeks prior to its delivery. They were asked to return an opt-out slip if they did not want their child to participate in a focus group to evaluate the intervention. Following delivery of the first *Operation Smoke Storm* session, teachers briefly outlined the purpose of the focus groups to students, and those interested in taking part were asked to write their name, gender and class on a piece of paper and hand this to the teacher. Students were informed that participants would be selected at random should more volunteer than needed. Students from School 2 were randomly selected from the list of volunteers by the PSHE teaching lead, whereas in School 1, volunteers’ names were handed to the research team who randomly selected students to take part. We planned to conduct four focus groups in each school, two for each gender, with up to 12 students in each, in line with recommendations on focus group size [18, 19]. Research suggests that smoking behaviours and attitudes differ according to gender [20, 21], and thus gender-specific groups were used both to encourage honest discussion and to allow us to explore any potential differences according to gender.

All teachers who delivered *Operation Smoke Storm*, along with the PSHE teaching lead from each school, were invited via e-mail to take part in a one-to-one face-to-face or telephone interview following delivery of all the sessions.

### Focus group and interview procedures

Three separate semi-structured discussion guides were developed based on existing literature and the content of the intervention, and agreed through discussion within the research team: one for the focus groups with students, another for interviews with teachers and one for PSHE teaching leads. The student focus group and teacher interview guides covered views on the acceptability and perceived effectiveness of *Operation Smoke Storm*, and the interview guide for the PSHE teaching leads also sought information about how PSHE (and specifically the topic of smoking) is usually taught in the school, by whom, and using what resources. There is evidence that follow-up ‘booster’ sessions are useful to strengthen and maintain the effectiveness of an intervention, and additionally that a wider approach tackling individual as well as family, community and societal influences is more likely to succeed in preventing young people from taking up smoking [8]. Therefore, the topic guides also sought students’ and teachers’ opinions on the potential acceptability and effectiveness of different options for a booster session for delivery in Year 8 and a family component to be delivered alongside the Year 7 resource.

Before the commencement of focus groups and interviews, participants were informed that data would be anonymised, treated confidentially and that they were free to withdraw at any point. Following this, written informed consent was obtained. Focus group discussions took place in a private room in both schools during lesson time, were facilitated by JT or AT (both experienced in facilitating qualitative research) with MB, LS or LJ acting as observers, and lasted 35 minutes on average (range 27 to 50 minutes). Face-to-face interviews also took place in a private room at the respective schools (conducted by JT or AT), and telephone interviews were conducted in a private room at Nottingham City Hospital (by AT) and lasted 34 minutes on average (range 24 to 50 minutes). All discussions were digitally audio-recorded.

### Data analysis

An external specialist transcription company transcribed interviews and focus group recordings clean verbatim. Transcripts were checked for accuracy (by JT and AT) and any potential identifiers were removed. Transcripts were assigned a unique code that identified the school (1 or 2) and focus group (male or female) or teacher number. Data were analysed using the framework approach [22, 23]. As an initial step to aid familiarisation, data from the first four focus groups and four teacher interviews were read several times by JT, AT and MB who independently summarised the data (using Microsoft Excel) and identified initial codes, themes and sub-themes in the data. This stage also enabled the researchers to ascertain whether there were any contradictory cases or any within- or between-group differences (according to school and gender). It was apparent that codes identified from both the focus groups and teacher interviews were similar (apart from teachers’ interview data identifying a theme specifically about preparation to deliver the resource) and thus data were analysed together. Codes, themes and sub-themes were subsequently discussed between the researchers resulting in an initial analytical framework. The framework was then applied and refined following analysis of the remaining transcripts which were divided between JT and AT. Data were then indexed according to the final framework using NVivo 10 software. Finally, transcripts were charted according to each theme to facilitate synthesis and interpretation. Data presented reflect the overall views of the participants from both schools.

## Results

We conducted eight focus groups in total (four at each school) with 79 students (39 males, 40 females) and an average of 10 students per group (range 8 and 11). Of the 23 eligible teachers, 18 were interviewed face-to-face and two by telephone. Three teachers (all from School 2) declined to take part due to time constraints.

Four core themes were identified and interpreted within the data: 1) teachers’ preparedness and delivery of *Operation Smoke Storm;* 2) raised awareness; 3) students’ engagement with *Operation Smoke Storm*; and 4) options for developing *Operation Smoke Storm*.

### Teachers’ preparedness and delivery of operation smoke storm

Both schools reported that it was their usual practice to cover the topic of smoking within the Year 7 PSHE curriculum, including the dangers of smoking and how to resist peer pressure to smoke; School 2 also included the influence of celebrity and role model smoking in their teaching. *Operation Smoke Storm* replaced these lessons. Although most of the teachers had no prior training or experience in teaching students about smoking (the majority were newly-qualified), after looking over the resource most felt confident about delivering *Operation Smoke Storm*. The ‘off-the-shelf’ nature of the resource, and that background knowledge about the topic or lengthy preparation time was not required, appealed to most teachers (Table 2, quote a). Some teachers spent more time preparing for the lessons than others, for instance by doing further independent research into the topic. Being given an appropriate amount of information and a training session prior to using the resource were seen to be important, and these helped to put a few teachers who were less confident at ease (Table 2, quote b).

**Table 2:** Teachers’ preparedness and delivery of Operation Smoke Storm

a) *I felt very good about it…everything was in place, that was brilliant.* (School 2, Teacher 2)
b) *The initial session [training session], yeah. I think that was definitely useful in terms of setting it up. It would have been quite difficult in my opinion; otherwise if we didn’t know the concept and the ideas behind it, I think that helped in terms of delivering it.* (School 1, Teacher 5)
c) *Some of the discussions we cut down quite a lot … it would have been lovely to have had more time.* (School 1, Teacher 4)
d) *There needs to be a bit more of, a teacher can override what is happening … so that it can suit the class. (*School 1, Teacher 7)
e) *The main thing would be a back button, without a shadow of a doubt.* (School 2, Teacher 3)
f)* I felt like I had to do a bit of research on my own, which wasn’t ideal, but if we had an information pack or something that said to us these are the kinds of things you’re going to come across*. (School 1, Teacher 2)

Teachers at School 1, where lessons were 40 minutes in length, found it very challenging to fit everything in, reporting specifically that there was insufficient time for class discussion to consolidate learning (Table 2, quote c). One teacher suggested that it would have been helpful to have been given more guidance on how to split the sessions up to enable delivery in lessons of varying length. Furthermore, teachers would have liked the functionality to easily navigate back and forth to recap if required, or skip sections to fit their lesson lengths (Table 2, quotes d and e).

A few students expressed concern to their teachers about family members who smoked and teachers’ confidence in dealing with these concerns varied. Some felt their skill as a teacher and the school procedures aided them through the process, but a lack of background knowledge and issues such as not knowing whether they gave the correct advice to students were raised by less confident teachers. They suggested that the provision of guidance on steering discussions, advice to give students, and signposts to background information on the topic would be helpful to counter this (Table 2, quote f).

### Raised awareness

Although many students mentioned that they knew a little bit about the harms of smoking and cigarette ingredients before beginning *Operation Smoke Storm*, the resource seemed to add more depth to their awareness (Table 3, quote a). For instance, students mentioned that they learnt new information about what is in a cigarette, such as chemicals also found in rat poison and petrol. Some students reported thinking, having learnt about cigarette ingredients and the health effects of tobacco use, that smoking was worse than they previously thought. A few students also reported having learnt about the nature of the tobacco industry, such as its focus on profits (Table 3, quote b), and how they target young people (Table 3, quote c).

**Table 3:** Raised awareness

a) *Back at primary school I did about three lessons on it, but when we did this it gave a lot more detail showing you what not to do and what was in it, so we can see how dangerous it was.* (School 1, M)
b)* They only do it just to make money; they don’t really care if people die.* (School 1, F)
c) *I’ve learnt that they try and make different kind of flavoured cigarettes to get different people, like they made, they tried testing chocolate flavoured cigarettes to get like young kids to smoke.* (School 2, F)
d)* I didn’t want to smoke to start with…but now I know I definitely, definitely don’t want to smoke.* (School 2, F)

Some students stated that participation in *Operation Smoke Storm* strengthened or maintained their aversion to smoking (Table 3, quote d) and felt it would for other young people too. However, a minority of students thought the resource may trigger thoughts about smoking amongst those who had not considered it previously.

### Students’ engagement with operation smoke storm

Students enjoyed the interactive nature of *Operation Smoke Storm,* which they reported was different to the usual format of their PSHE lessons. Generally, teachers felt the resource was appropriate for the age group and allowed all students to actively participate in the lessons (Table 4, quote a). Students mentioned that they liked the secret agent theme and teachers felt this was important in capturing the students’ interest and reported that the storyline engaged them throughout. However, a couple of teachers reported that their class did not relate well to the storyline but that this did not impact upon their engagement in the lessons.

**Table 4:** Students’ engagement with Operation Smoke Storm

a)* I liked how it was more on them, it wasn't me at the front just talking to them. It was them watching stuff and then answering questions about it. So it really got them involved.* (School 2, Teacher 5)
b)* You can use your imagination to create it and make it what you want to make it.* (School 2, M)
c)* I’ve got a couple of kids in my class who are dyspraxic and dyslexic, because there’s not a lot of reading it was really good for them, they liked the tick box stuff, but then when we got to this section of ‘remember all this information and now create your own’, they found that more difficult because they hadn’t retained the information as well.* (School 1, Teacher 1)

Having a variety of activities to take part in seemed to maintain students’ engagement and they enjoyed learning from peers in group work and the creative freedom they had when putting together group presentations (Table 4, quote b). One activity required students to recall facts from a previous lesson and some students found this difficult. Teachers suggested including a plenary session or providing teacher answer sheets would be useful ways to counteract the problem.

Some teachers highlighted that aspects of the resource could be improved to better cater for lower-ability students who struggled with remembering facts (Table 4, quote c) and felt that some differentiation in the activities could be beneficial. However, they acknowledged that the absence of differentiation did not impede students’ ability to engage with the resource.

### Options for developing operation smoke storm

Both students and teachers supported the idea of a ‘booster’ session delivered a year later when students are in Year 8 (aged 12-13 years) to reinforce learning about how the tobacco industry targets young people. In general, teachers felt that a classroom session would work better than a homework activity, and they suggested the booster should include a reminder of *Operation Smoke Storm* followed by a progressive activity to cater for students’ growing maturity (Table 5, quote a). Students were asked to consider the acceptability and potential effectiveness of playing a game (either paper-based, such as a board game, or an electronic game on a computer, tablet or mobile telephone) or producing their own short film, where the example of *Cut Films* [24] was presented to students in focus groups; both options were well received by students and they suggested that an anti-smoking message delivered by peers of their own age might be effective (Table 5, quote b). However, though teachers liked the ideas they felt that both would be difficult to deliver in school for logistical reasons, such as not having enough equipment (tablets, computers or video recorders) or space (computer rooms) to accommodate all classes at the same time (Table 5, quote c). Regardless of the content of a booster session, teachers were again in favour of a teacher-led ‘off the shelf’ resource similar to *Operation Smoke Storm.*

**Table 5:** Options for developing Operation Smoke Storm

a)* I don’t think that [secret agent undercover] would fly again. It would maybe have to be something a little bit different.* (School 1, Teacher 10)
b)* I think it will encourage other people who are watching it to not smoke because they’ll know that other people around their age are saying it and they’ll be persuaded more to not do it.* (School 1, M)
c)* They’re not allowed to bring mobile phones into school so they wouldn’t do it on their own phones. PSHE… doesn’t have tablets and… getting in the computer room is a bit of a nightmare.* (School 1, Teacher 7)
d)* Also some people’s parents work a lot, because my mum’s a nurse so she works nights, and it would be quite hard for me to get her to fill it out if she was working.* (School 2, F)
e)* The students don’t always … speak to their parents about what they’ve done in the school, so that’s a physical reminder of what they’ve done and there’s more opportunity for parents to engage.* (School 1, Teacher 5)

Students and teachers were also asked to think about the most effective ways to engage families in discussions around the anti-smoking message. In particular, participants were asked to comment on the idea of a take-home booklet containing information and activities for parents and guardians to complete with their child. In principle, students felt that this would be a good way of involving their families in their learning but opinion was mixed in terms of whether their parents would read it. Some also questioned whether their parents would have time to complete the activities (Table 5, quote d). Teachers expressed the importance of parental engagement in the students’ learning, but from prior experience stressed that this remained a challenge. Thus teachers were generally in favour of a booklet (Table 5, quote e), as long as it was carefully worded so as not to offend parents who themselves smoke.

## Discussion

This study reports the findings of a qualitative evaluation of the implementation of a novel tobacco control intervention in two schools. We found that the off-the-shelf resource was well received by students and teachers, providing novel lessons requiring little preparation on the teachers’ part whilst being engaging to students and increasing their awareness of smoking-related issues. Suggestions on how to improve the current resource were discussed, as well as ways to extend the resource with a booster session and involve families in discussions about smoking.

Our study addresses an identified need to investigate the relevance of prevention interventions based on awareness of tobacco industry tactics in school settings [8]. Our findings showed that students related well to the secret agent scenario and undercover investigation of the tobacco industry. Although the depth of discussion in focus groups with students was restricted by the limited time available, some students did report they had gained new knowledge about the tobacco industry from *Operation Smoke Storm*. Some reported that *Operation Smoke Storm* had strengthened or maintained their aversion to smoking, though focus groups were not able to distinguish whether it was the tobacco industry-related content of the intervention in particular which was responsible for this aversion. It is not possible to conclude from this cross-sectional study whether students’ increased awareness of tobacco industry tactics will reduce smoking uptake, though our planned full-scale cluster-randomised controlled trial will address this question.

Recent and robust qualitative evaluations of school-based smoking prevention interventions, as well as interventions for other unhealthy behaviours, are limited. Our findings are likely to have wider relevance for those planning to deliver and evaluate health promotion interventions in schools. The appeal of an ‘off-the-shelf’ resource, as highlighted by teachers in this study, is likely to apply not just to smoking prevention interventions but also to teaching on other topics within the remit of PSHE, such as alcohol and drugs education. In the UK in 2009, over half of all secondary schools had no members of staff with the national accredited qualification in PSHE education [25]. An intervention which teachers are able to deliver with little to no background knowledge or preparation is likely to be more acceptable.

Our findings also highlight the importance of school-based interventions being flexible and adaptable to variations in lesson length and resource availability. This may become increasingly important given that the amount of teaching time allocated to PSHE has generally declined in recent years, and the subject’s non-statutory status has led to some schools prioritising other ‘core’ subjects [26, 27]. Whilst both students and teachers welcomed the novel and engaging web-based resources in *Operation Smoke Storm*, they reported limited ability to provide individual student access to computer facilities. Other school-based interventions should not rely on this as a means of reaching and engaging students.

Whilst we conducted a rigorous qualitative assessment of *Operation Smoke Storm*, independent of those who developed and delivered the intervention, we acknowledge several limitations. Our findings are based on intervention delivery in just two schools and thus may not be transferable to other settings. However, similar themes were reported across both schools and thus we are confident that they are generalizable to some extent. We were restricted to conducting focus groups during lesson time (40 minutes and 60 minutes in length) and this limited our ability to explore certain aspects in depth. Even though students were randomly selected to take part in focus groups, findings may be biased as our sampling frame was those who were willing to participate; it is possible that those students who did not volunteer were more disengaged in the lessons and receiving an anti-smoking message.

## Conclusions

In conclusion, the evidence we have gathered from two schools suggests that *Operation Smoke Storm* is an acceptable smoking-prevention intervention for use by teachers with Year 7 students. Some changes will, however, now be made to the resource to improve its flexibility and ease of use with classes of different abilities, lesson length and teacher confidence and knowledge. A booklet for students to take home to prompt discussion between them and their parents or guardians will be developed to accompany the Year 7 *Operation Smoke Storm* intervention, as well as a booster session for use with students when they are in Year 8, again ensuring this is easy to use, engaging and matches the increased maturity of the students. The revised and extended intervention package will be piloted in the same two schools, with further qualitative and quantitative evaluation. If there is sufficient promise of effectiveness the intervention will be evaluated in a full scale cluster-randomised controlled trial.

## Abbreviation

PSHE, Personal, Social, Health and Economic Education
